# Agreement Between Venous and Capillary Haemoglobin Concentrations in a High-Burden Urban Indian Setting: A Cross-Sectional Analysis

**DOI:** 10.3390/diagnostics16203419

**Published:** 2026-10-21

**Authors:** Nandini Sharma, Saurav Basu, Mongjam Meghachandra Singh, Mrunali Zode, Shikha Taneja Malik, Shivani Rao

**Affiliations:** 1 Department of Community Medicine, SGT University, Gurugram 122505, India; hod.community@sgtuniversity.org; 2 Department of Community Medicine, ESIC Medical College and Hospital, Joka, Kolkata 700104, India; 3 Department of Community Medicine, Maulana Azad Medical College, New Delhi 110002, India; mm.singh22@gov.in; 4 Independent Researcher, New Delhi 122002, India; mzode04@gmail.com; 5 Drugs for Neglected Disease Initiative (DNDi), New Delhi 110016, India

**Keywords:** HemoCue, haemoglobin, haemoglobinometers, validation, anaemia, method-comparison

## Abstract

**Background**: Anaemia is a major public health issue in India, where point-of-care testing is central to national control strategies. This study evaluated the diagnostic accuracy of the HemoCue^®^ Hb 201^+^ for haemoglobin (Hb) estimation from capillary blood compared to the gold-standard venous blood analysis. **Methods**: In a cross-sectional study of 748 participants in a low-resource urban Delhi setting, Hb levels from single-drop capillary samples (HemoCue^®^ Hb 201^+^) were compared to those from venous samples (automated autoanalyzer). Agreement was assessed using Bland–Altman analysis, Passing–Bablok regression, and standard diagnostic accuracy metrics. **Results**: The mean (SD) haemoglobin concentration was lower in capillary samples (11.83 [1.95] g/dL) than in venous samples (12.56 [1.89] g/dL). Capillary Hb was, on average, 0.73 g/dL lower than venous Hb (95% limits of agreement: −3.11 to 1.65 g/dL). Consequently, the HemoCue^®^ Hb 201^+^ significantly overestimated anaemia prevalence (54.4% vs. 35.0%). The capillary method demonstrated high sensitivity (92.4%) for detecting anaemia but only moderate specificity (66.1%), indicating a tendency for false positives. **Conclusions**: Although the HemoCue^®^ Hb 201^+^ is a sensitive tool for anaemia screening in field settings, the moderate specificity and the significant negative bias can lead to overestimation of prevalence and potential misclassification of individuals. However, the limitation of single-drop capillary sampling warrant cautious interpretation of results, particularly for clinical decision-making and precise prevalence estimation.

## 1. Background

Anaemia remains a significant global health challenge, affecting one-fourth of the world’s population. The burden is disproportionately borne by children under five, women, and populations in sub-Saharan Africa and South Asia [1]. In response, the World Health Organization (WHO) has set a target to reduce anaemia among women of reproductive age by 50% by 2025 as part of its global nutrition agenda [2].

Despite concerted government efforts, India has one of the highest burdens of anaemia globally. According to the National Family Health Survey (NFHS-5), 67% of children aged 6–59 months, 57% of women, and 25% of men aged 15–49 years are anaemic [3]. To address this, the Indian government launched the Anaemia Mukt Bharat initiative, which promotes rapid, point-of-care screening for anaemia in vulnerable populations [4].

Haemoglobin (Hb) concentration is the key biomarker for diagnosing anaemia. Although venous blood analysis using automated haematology analyzers is the gold standard, this method is resource-intensive and often unfeasible for large-scale surveys in low-resource settings. Consequently, capillary Hb measurement using portable point-of-care devices, such as HemoCue, offers a practical alternative for rapid screening. However, despite their widespread use for decades, recent evidence has raised concerns about their accuracy, citing large random variations in test results [5,6].

HemoCue devices are widely used due to their portability, speed, and ease of use, but evidence comparing their accuracy to laboratory analyzers is mixed. Some studies report that capillary Hb estimates are comparable to venous measurements [7,8,9], while others highlight significant deviations that demand cautious interpretation [10,11,12,13,14]. Pre-analytical factors, such as sample collection timing and technique, can further influence results [15].

Studies comparing capillary and venous Hb concentrations have reported variable findings. Neufeld et al. found that capillary Hb was, on average, 0.5 g/dL higher than venous Hb in both children and adults [16]. Similarly, Namaste et al. and Hinnouho et al. reported higher Hb concentrations in pooled and single-drop capillary samples, respectively, compared to venous samples, resulting in lower estimates of anaemia prevalence [17,18]. In contrast, a study in Uttar Pradesh found that venous Hb was, on average, 0.9 g/dL higher than capillary Hb, leading to a higher anaemia prevalence when using capillary samples [19].

These conflicting findings underscore the need for careful evaluation of point-of-care devices, as their results are pivotal in shaping national health policies, guiding resource allocation, and informing anaemia control programs. Therefore, this study aimed to compare Hb measurements from capillary blood using the HemoCue^®^ Hb 201^+^ with those from venous blood analyzed by an automated haematology analyzer in a low-resource urban setting in India and to assess the implications of these findings for clinical and public health practice.

## 2. Methodology

**Design and participants**: This cross-sectional analysis was conducted within a larger cohort study in an urban resettlement and slum area of North-East Delhi, a Demographic, Developmental, and Environmental Surveillance Site (DDESS) with an estimated population of 50,000 during 2023–24. The site was selected to represent a resource-limited setting with a low-income sociodemographic profile.

**Sample size and sampling**: The study included 748 participants selected through a multistage sampling process. As part of the parent cohort study, the site was divided into five equal-sized population clusters. A line list of all residents was generated from the site’s census database. Participants were then enrolled from these clusters using an age-proportionate sampling technique that mirrored the community’s age distribution (2–4 years: 5%; 5–9 years: 8%; 10–19 years: 18%; 20–49 years: 51%; 50–64 years: 12%; ≥65 years: 4%). Enrollment was conducted via consecutive house-to-house visits.

**Data collection**: Trained field investigators recruited the participants, and data were entered into a predesigned questionnaire through electronic paperless data collection on android tablets using proprietary software. A total of 5 mL venous blood was collected by three trained lab technicians cum phlebotomists (one male and two females, with each having prior experience in pediatric phlebotomy) through venipuncture under aseptic precautions in K2 EDTA vials for haemoglobin measurement (Central Blood Count test) using the Sysmex XN-1000 (Sysmex Corporation, Kobe, Japan) autoanalyzer. The samples were transported in chiller boxes equipped with automated temperature loggers while maintaining a consistent cold chain (2–8 °C) to a central NABL-accredited and ISO 15189:2022 (Geneva, Switzerland) compliant laboratory on the same day of sample collection.

Capillary blood was used to assess Hb using the HemoCue^®^ Hb 201^+^ system. Blood collection with HemoCue was ensured through adherence to WHO-recommended best practices. A single-use sterile lancet was used to make a clean puncture, and the initial drop of blood was discarded, and the second drop was used for the analysis. If blood flow was slow, gentle, intermittent pressure was applied away from and above the puncture site, carefully avoiding any forceful squeezing [20].

Quality assurance: To ensure data quality, approximately 5% of sample collections were directly supervised. The HemoCue^®^ Hb 201^+^ system underwent internal quality control checks daily before use. The external quality control calibration was performed by an external supervising agency at the initiation of the study, and subsequently on monthly intervals or after every 100 samples, whichever was earlier.

### Statistical Analysis

Descriptive statistics were used to summarize participant characteristics. The agreement between venous and capillary Hb measurements was assessed using a Bland–Altman plot with 95% limits of agreement (LoA), Passing–Bablok regression, and Lin’s Concordance Correlation Coefficient (CCC). A bias between 0 and 0.5 g/dL was predefined as clinically acceptable, as a difference within this range is unlikely to alter anaemia severity grading or treatment plans in primary care, except at a diagnostic threshold [16,21].

Participants were classified as anaemic or non-anaemic according to WHO age- and gender-specific Hb cutoffs. McNemar’s test was used to assess for significant differences in anaemia classification between the two methods, and the kappa statistic was calculated to measure the degree of agreement. The sensitivity, specificity, positive predictive value (PPV), and negative predictive value (NPV) of the capillary method were calculated using the venous method as the reference standard. A Receiver Operating Characteristic (ROC) curve was generated to evaluate the diagnostic ability of the capillary method.

Finally, a multivariable linear regression model was fitted to identify factors associated with the difference between venous and capillary Hb measurements. The dependent variable was the difference (venous Hb-capillary Hb), and independent variables included age, gender, BMI, and a history of chronic medical conditions. A *p*-value < 0.05 was considered statistically significant. All analyses were performed using Stata version 15.1 (StataCorp, College Station, TX, USA).

**Ethical considerations:** The present study conducts secondary analyses on additional haemoglobin estimation data collected as part of a serosurvey that was approved by the Institutional Ethics Committee, Maulana Azad Medical College, New Delhi. Written and informed consent was taken from all adult participants, and assent and parental consent from minors aged above 7 years. Parental consent alone was obtained for children aged below 7 years.

All participants were informed that the study involved anaemia screening using two methods (finger-prick and venous draw) to compare the point-of-care device with laboratory results. They were told that the findings would help clarify the utility of the HemoCue^®^ system in community settings.

All participants received their laboratory test results. Clinical decisions regarding anaemia status were based on the venous sample results. Participants diagnosed with mild to moderate anaemia were provided with iron and folic acid supplements and dietary counselling. Those with severe anaemia were counselled and referred to a government tertiary care hospital for further management.

## 3. Results

This study included 748 participants, aged 2–83 years, whose haemoglobin (Hb) levels were measured using both capillary and venous methods. The mean (SD) age was 31.45 (17.76) years, with 78.5% of participants over 15 years old. The sample included children aged 2–4 years (4.3%), 5–11 years (11.5%), and adolescents aged 12–14 years (5.8%). A slight majority of participants were female (56.82%). Chronic medical conditions were reported by 13.5% of participants. Key demographic characteristics included Hindu religion (97.06%), Scheduled Caste status (55.29%), and 6 to 10 years of schooling (33.82%) (Table 1).

The mean (SD) Hb concentration was significantly lower in capillary samples (11.83 g/dL [1.95]) than in venous samples (12.56 g/dL [1.89]), with a mean difference of −0.73 g/dL (SD 1.19; *p* < 0.001) (Figure 1, Table 2). The 95% limits of agreement ranged from −3.11 g/dL to 1.65 g/dL, indicating considerable variability between the methods. Despite this, Lin’s concordance correlation coefficient (CCC) analysis demonstrated moderate agreement between the two measures (CCC = 0.75, precision = 0.80, accuracy = 0.93) (Table 3, Figure 2).

**Figure 1:**
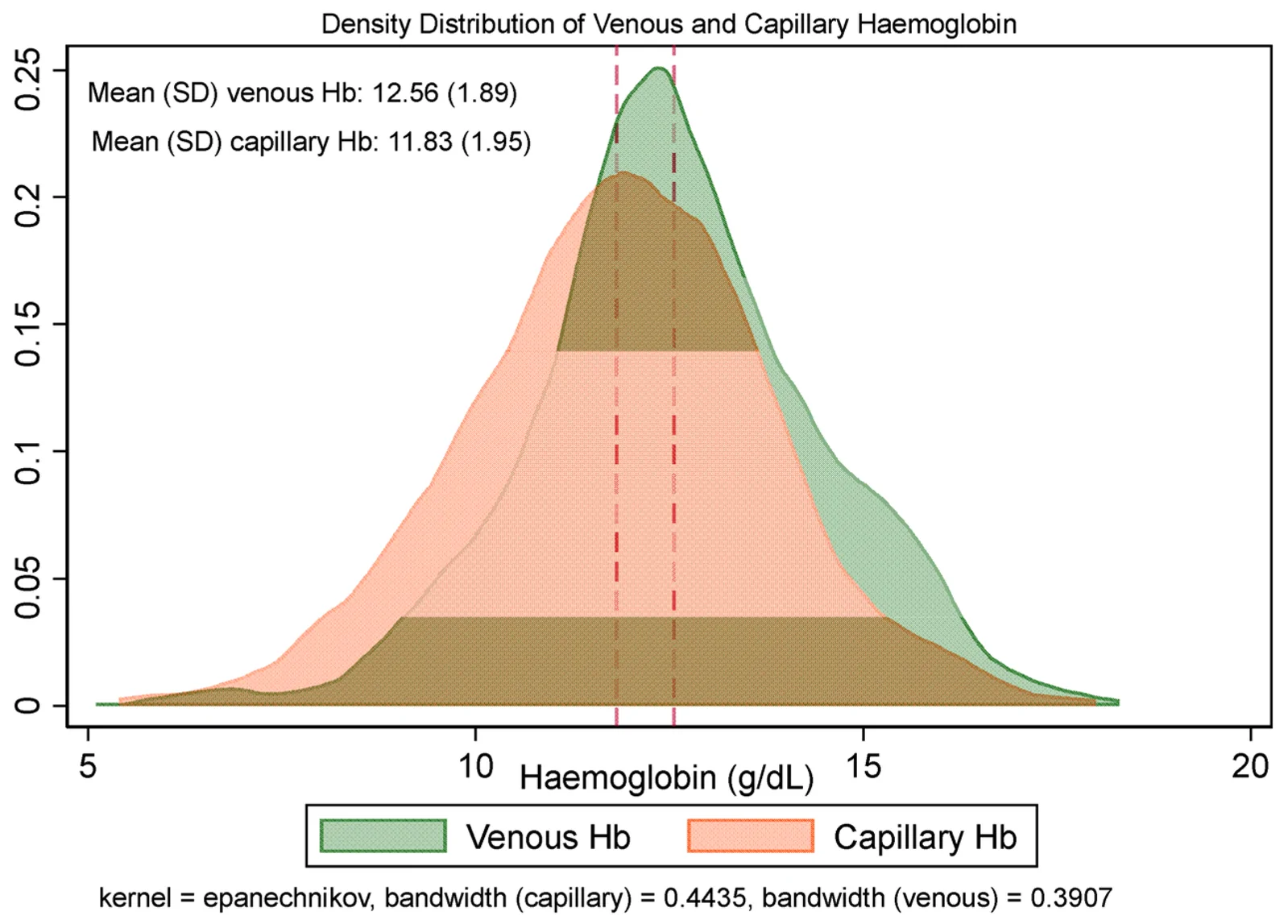
Density distribution plot of haemoglobin measurements using capillary (HemoCue^®^ Hb 201^+^) and venous samples (laboratory autoanalyzers).

**Figure 2:**
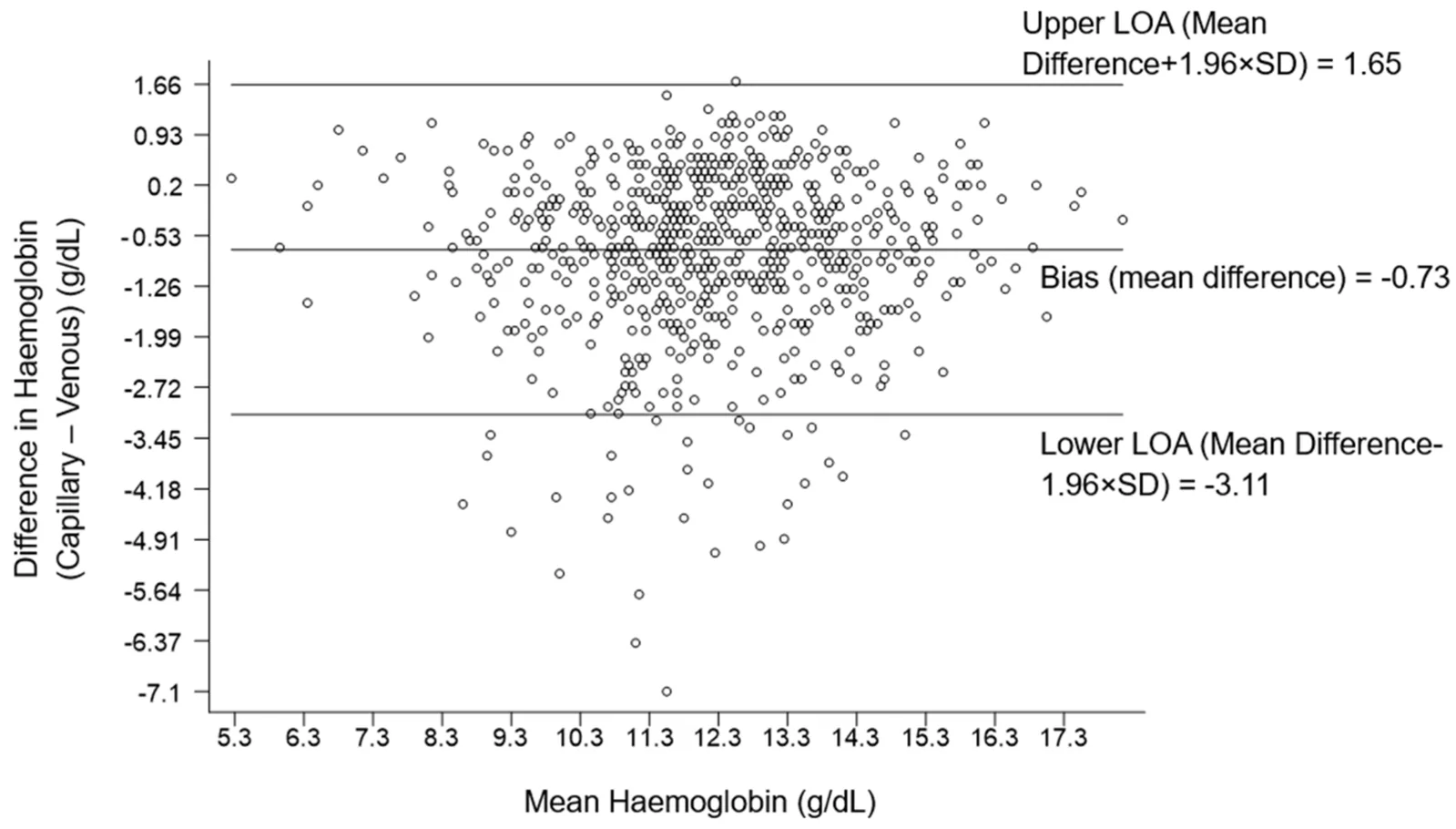
Bland–Altman Plot for agreement between capillary (HemoCue^®^ Hb 201^+^) and venous Hb measurements (laboratory autoanalyzers).

Passing–Bablok regression analysis revealed a constant negative bias, with capillary Hb readings being, on average, 0.94 g/dL lower than venous Hb (intercept = −0.94, 95% CI: −1.55 to −0.60). The slope of 1.03 (95% CI: 1.00 to 1.08) suggested proportional agreement, although the confidence interval approaching 1.00 indicates this was not conclusive. Residual analysis highlighted greater variability at extreme Hb concentrations. The statistically significant CUSUM test (*p* < 0.01) confirms that the relationship between capillary and venous measurements is non-linear. Residual analysis indicates that while agreement is relatively stable in the middle of the physiological range, the discrepancy between methods widens at the extremes (Figure 3). Specifically, at lower hemoglobin concentrations, the capillary method tends to further underestimate Hb, while at higher concentrations, the variance increases, leading to less predictable results.

The prevalence of anaemia was substantially higher when measured with capillary samples (54.41%; 95% CI: 50.76% to 58.02%) compared to venous samples (35.03%; 95% CI: 31.61% to 38.45%). Using venous measurements as the reference standard, the capillary method demonstrated a sensitivity of 92.37% and a specificity of 66.05% for diagnosing anaemia. The methods showed moderate agreement (75.27% overall agreement; Kappa = 0.52, *p* < 0.001) (Table 4).

In the subgroup of children (up to 14 years), the mean difference (SD) between capillary and venous Hb was −0.22 (1.05) g/dL, with 95% limits of agreement from −2.32 to 1.87 g/dL. For diagnosing anaemia in this group, the HemoCue^®^ Hb 201^+^ showed moderate sensitivity (70.83%; 95% CI: 63.81% to 77.85%) and specificity (80.29%; 95% CI: 74.15% to 86.44%). The positive predictive value was low (38.64%; 95% CI: 31.12% to 46.16%), while the negative predictive value was high (94.02%; 95% CI: 90.35% to 97.68%). The kappa statistic (ĸ = 0.38, *p* < 0.001) indicated fair agreement, though the risk of misclassification, particularly false positives, was notable (Supplementary Tables S1 and S2).

Furthermore, to better understand measurement agreement near diagnostic cut-offs for anaemia, a focused analysis was conducted for participants whose venous Hb values lie close to the WHO-defined thresholds (between 8–13 g/dL), separately for males aged 15 years or older and non-pregnant women aged 15 years or older. In this range, the mean difference (bias) between capillary and venous Hb was −0.87 g/dL (95% LoA: −3.43 to 1.69) for men (*n* = 38) and −0.65 g/dL (95% LoA: −2.68 to 1.38) for non-pregnant women (*n* = 301). While sensitivity for detecting anaemia using the HemoCue device was high (93.8% in men, 94.3% in women), specificity was low (33.3% and 42.2%, respectively), indicating a higher likelihood of false-positive anaemia classification near the diagnostic cut-offs. The agreement between methods was only fair (Cohen’s κ = 0.31 for men, κ = 0.40 for women), indicating that 16 to 25% of individuals in this range would be misclassified by the capillary method. (Supplementary Tables S3 and S4).

McNemar’s test confirmed a significant difference in anaemia prevalence between the two methods, a finding that was consistent across all subgroups: children up to 14 years (*n* = 161), non-pregnant women aged 15 and over (*n* = 348), and men aged 15 and over (*n* = 232). Despite these differences, the capillary method demonstrated strong diagnostic utility in distinguishing between anaemic and non-anaemic individuals, with an area under the receiver operating characteristic (ROC) curve of 0.88 (95% CI: 0.86 to 0.91) (Figure 4).

Regression analysis revealed that both age and gender were significant predictors of the difference between venous and capillary Hb measurements. The discrepancy between methods increased with age (β = 0.013 per year, 95% CI: 0.007 to 0.02). The difference was also significantly smaller for females compared to males (β = −0.29, 95% CI: −0.47 to −0.12) (Table 5).

## 4. Discussion

The current study assessed the agreement between haemoglobin (Hb) measurements from capillary blood samples using the HemoCue^®^ Hb 201^+^ and those from venous samples using laboratory autoanalyzers. On average, capillary Hb values were 0.73 g/dL lower than venous values, and this mean bias exceeded the predefined clinical acceptance threshold of 0–0.5 g/dL. The device demonstrated high sensitivity in detecting anaemia but only moderate specificity, suggesting a tendency for false-positive results. However, the finding should be interpreted cautiously due to the use of the single-drop capillary sampling technique which is a known driver of increased variability and lower Hb levels compared to pooled or venous samples.

These findings are consistent with previous research [11,21,22,23]. For instance, two studies from India reported that capillary Hb, measured from a single drop with HemoCue devices, was consistently lower than venous Hb measured by a reference autoanalyzer, thereby overestimating the prevalence of anaemia [22,23]. One of these studies involved blood donors at a tertiary hospital in Uttarakhand [23], while the other included 102 pregnant women with a mean age of 23.3 ± 3.4 years at antenatal clinics in Haryana [22]. Similarly, Karakochuk et al. observed a higher anaemia prevalence when using capillary samples with the HemoCue^®^ Hb 201^+^ (41.0%) compared to venous samples processed by automated analyzers (29.5%) in Cambodian women aged 18–45 years [11]. Caulfield et al. also reported that single-drop capillary Hb values from the HemoCue^®^ Hb 201^+^ in late-pregnancy women aged 18–40 were lower than true Hb levels and showed considerable variability, raising concerns about the device’s accuracy [21]. Furthermore, research in Indian field settings has shown only moderate statistical agreement between HemoCue measurements and those from dried blood spots [24].

Conversely, some studies have reported capillary Hb values from the HemoCue^®^ Hb 201^+^ to be higher than venous values from automated analyzers [25,26,27,28]. Patel et al. found that healthy blood donors aged 20 to 71 years had higher capillary Hb levels measured with the HemoCue^®^ Hb 201^+^ compared to venous samples analyzed with the Cell-Dyn 4000 analyzer [25]. These conflicting results highlight the influence of population characteristics, physiological conditions, and differences in sampling and analysis methods on haemoglobin measurements.

An important consideration when interpreting these results is the sampling method, as evidence indicates significant differences between single-drop and pooled capillary blood measurements. Studies have shown that single-drop samples often yield lower Hb concentrations and greater variability than pooled samples, leading several authors to caution against their use [12,17,29,30,31]. In a recent multisite study, the 95% limits of agreement (LOA) for HemoCue measurements were narrowest for venous samples (±4.8–16 g/L), followed by pooled capillary (±8.6–28.6 g/L), and widest for single-drop capillary samples (±9.4–36.8 g/L), with some single-drop discrepancies reaching 37 g/L [31].

In our study, the wide 95% limits of agreement (–3.11 to 1.65 g/dL) underscore the considerable variability between the two methods. This suggests that while capillary measurements with the HemoCue device are useful for rapid screening, they may not be interchangeable with results from laboratory autoanalyzers. Such variability could lead to incorrect classifications of anaemia severity, a concern also noted by Larson et al. when evaluating capillary Hb measurements in children for population-level assessments [32]. Similarly, the low positive predictive value (38.64%) among children observed in this study highlights a high risk of false-positive anaemia classification. Overdiagnosis in this vulnerable group can lead to unnecessary iron supplementation and associated side effects, while triggering heightened parental anxiety. Furthermore, the resulting need for confirmatory venous testing is likely to increase the burden on healthcare systems which is particularly paradoxical in resource-limited settings where these devices are most frequently deployed due to limited laboratory infrastructure and high turnaround times.

Furthermore, we observed a higher prevalence of anaemia with the capillary method than with the venous method. This discrepancy is significant and highlights the potential clinical implications of using different sampling techniques. The high sensitivity and moderate specificity of the capillary method suggest that while it effectively identifies individuals with anaemia, it may also lead to overdiagnosis. This aligns with findings from other studies that have noted the tendency of capillary measurements to overestimate anaemia prevalence [11,26].

The subgroup analysis near diagnostic thresholds quantifies the device’s dual nature. Within this critical range, the observed 0.73 g/dL negative bias and wide limits of agreement result in the misclassification of individuals with borderline-normal haemoglobin as anaemic, or the over-grading of anaemia severity according to WHO cutoffs. These reclassifications directly alter clinical pathways for patients who would be categorized as normal by gold-standard venous analysis. Consequently, the device’s underestimation within these borderline zones significantly compromises the precision of individual anaemia assessment. While the HemoCue’s high sensitivity supports its utility for ruling out anaemia in community screening, its poor specificity in the diagnostic borderline zone necessitates confirmatory venous testing for accurate individual clinical management. Furthermore, at the lower end of the hemoglobin range, the increased underestimation bias likely increases the frequency of ‘false-positive’ severe anaemia triggers which can lead to a higher volume of unnecessary clinical referrals. For public health programs, these findings underscore that capillary-based prevalence estimates should be interpreted as screening figures subject to a predictable overestimation bias, rather than as definitive diagnostic rates for guiding individual treatment pathways.

This investigation was part of a larger cohort study on dengue and chikungunya prevalence and was not specifically powered to assess the agreement between Hb measurement methods, which may have limited the precision of our findings. Nevertheless, the inclusion of 748 participants provided high statistical precision with a resultant standard error of 0.015 g/dL, with narrow 95% confidence intervals for both the mean bias (−0.82 to −0.65 g/dL) and the limits of agreement (LoA) (3.26 to −2.97 g/dL for the lower LoA, and 1.51 to 1.80 for the upper LoA). Because this margin of error is substantially smaller than our predefined clinical threshold of 0.5 g/dL, the study was sufficiently powered to confirm that the observed discrepancies are both statistically significant and clinically meaningful.

Additionally, the regression model used to explore factors contributing to the measurement variability was limited in scope. Furthermore, the available data could not account for several biologically plausible factors that can influence capillary haemoglobin measurements, such as hydration status, acute infection, peripheral perfusion, or recent physical activity. These variables are known to affect peripheral blood flow and plasma volume, potentially confounding the observed associations with age and gender [33,34]. While our study identifies demographic trends in anaemia measurement, these findings may be partially mediated by these unmeasured individual physiological and environmental determinants.

Another limitation is our use of a single (the second) drop of capillary blood instead of a pooled sample, a constraint of the field setting that could have introduced variability and affected measurement precision.

In conclusion, the HemoCue^®^ Hb 201^+^ is a sensitive tool for anaemia screening in field settings. However, the moderate specificity and significant negative bias observed may lead to overestimation of prevalence and potential misclassification. These findings likely reflect the interplay between device performance and the methodological limitations of single-drop capillary sampling, warranting a cautious interpretation of results, particularly for clinical decision-making and precise prevalence estimation

The present findings underscore the need to recognize both the strengths and limitations of capillary Hb measurements from devices like the HemoCue^®^ Hb 201^+^, particularly for individuals whose values are near clinical decision thresholds. Although the observed bias exceeded the predefined acceptance limit, this modest deviation could affect diagnostic accuracy, especially in borderline anaemia cases. Nevertheless, the device’s operational advantages—portability, rapid results, and minimal training requirements render it a valuable tool for large-scale anaemia surveillance, particularly in resource-constrained settings. However, clinicians and public health practitioners should interpret results cautiously where precise anaemia prevalence is critical for patient care or health policy. For clinical decision-making or in studies requiring precise Hb quantification, confirmatory testing with reference laboratory methods remains essential.

Future research should focus on refining capillary sampling techniques and identifying factors that contribute to the observed discrepancies. Standardizing sample collection, improving device calibration, and conducting population-specific validations could enhance the reliability and accuracy of capillary haemoglobin measurements.

## Figures and Tables

**Figure 3:**
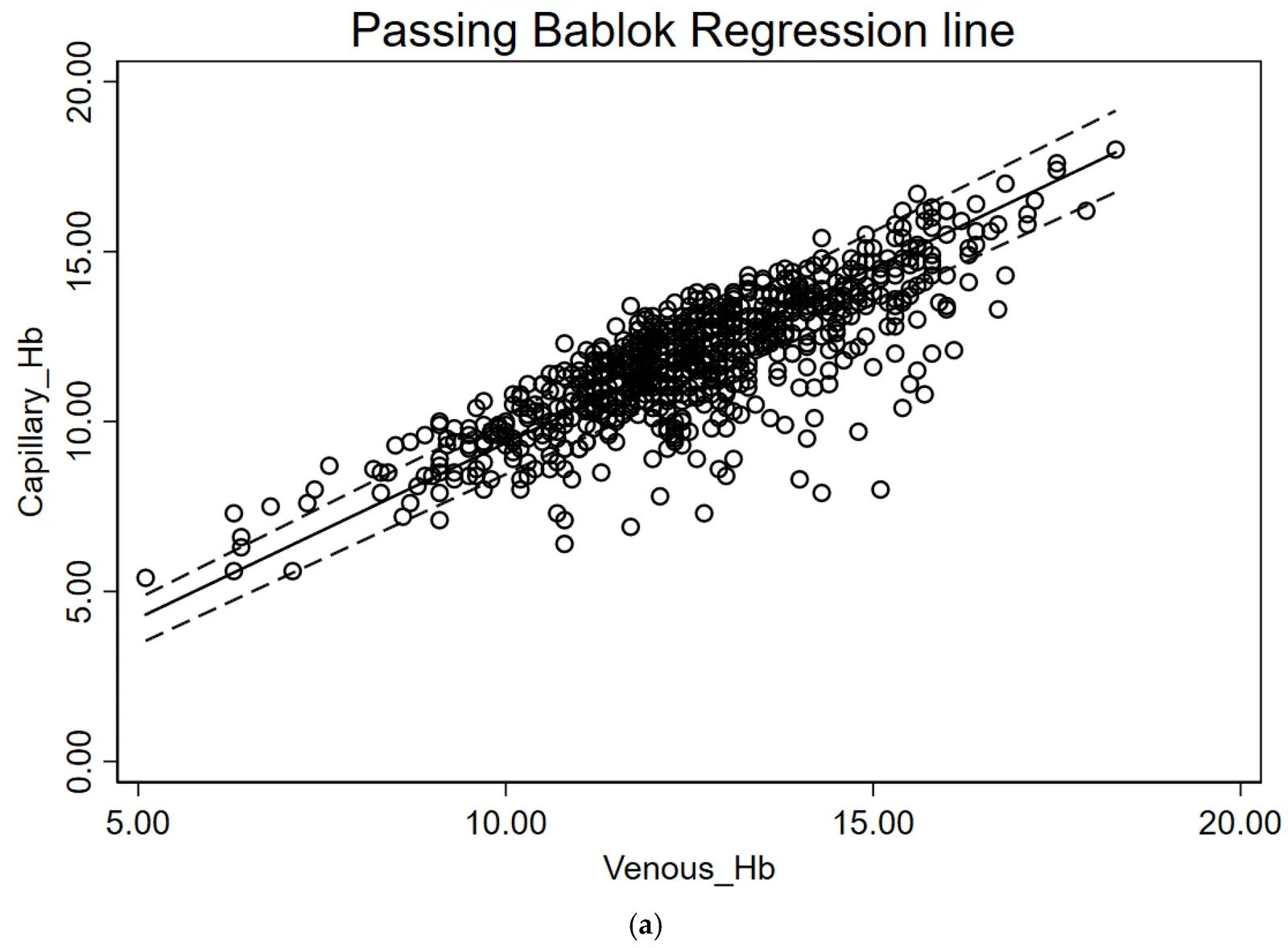

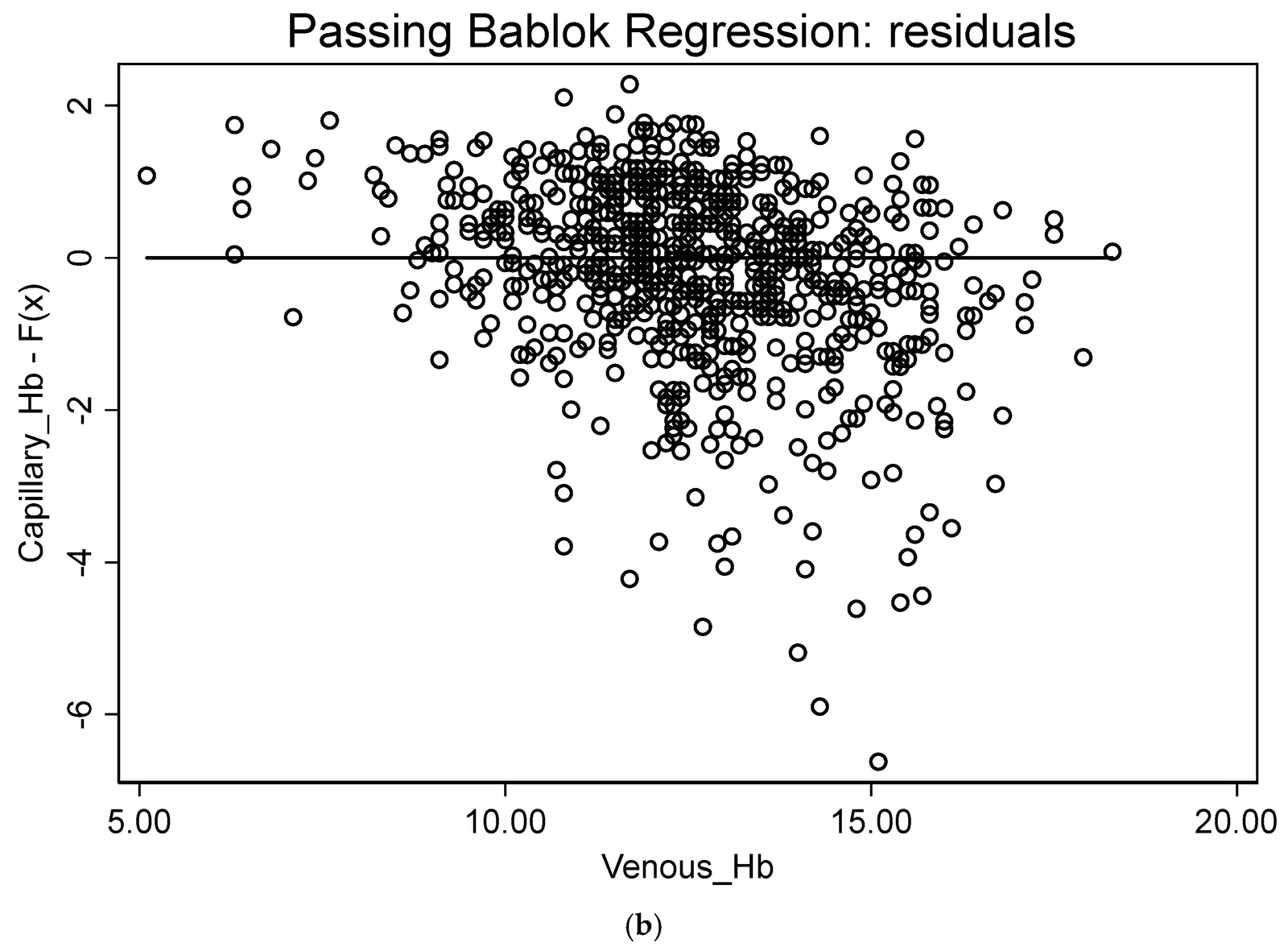
(**a**): Passing–Bablok Regression Plot–Regression line (capillary vs. venous Hb measurements in g/dL). Passing–Bablok Regression Line: Capillary Hb (g/dL) = Intercept + Slope × Venous Hb (g/dL). Passing–Bablok Regression Line: Intercept: −0.94 (95% CI: −1.55, −0.60); Slope: 1.03 (95% CI: 1.00, 1.08); CUSUM Test for deviation from linearity: *p*-value between 0.001 and 0.01. (**b**): Passing–Bablok Regression Plot–Residuals. Residual (*Y*-axis) = Capillary Hb (measured) − F (Venous Hb). F (Venous Hb) is predicted capillary Hb = Intercept + Slope × Venous Hb (g/dL). A horizontal line at 0 represents the line of perfect agreement between.

**Figure 4:**
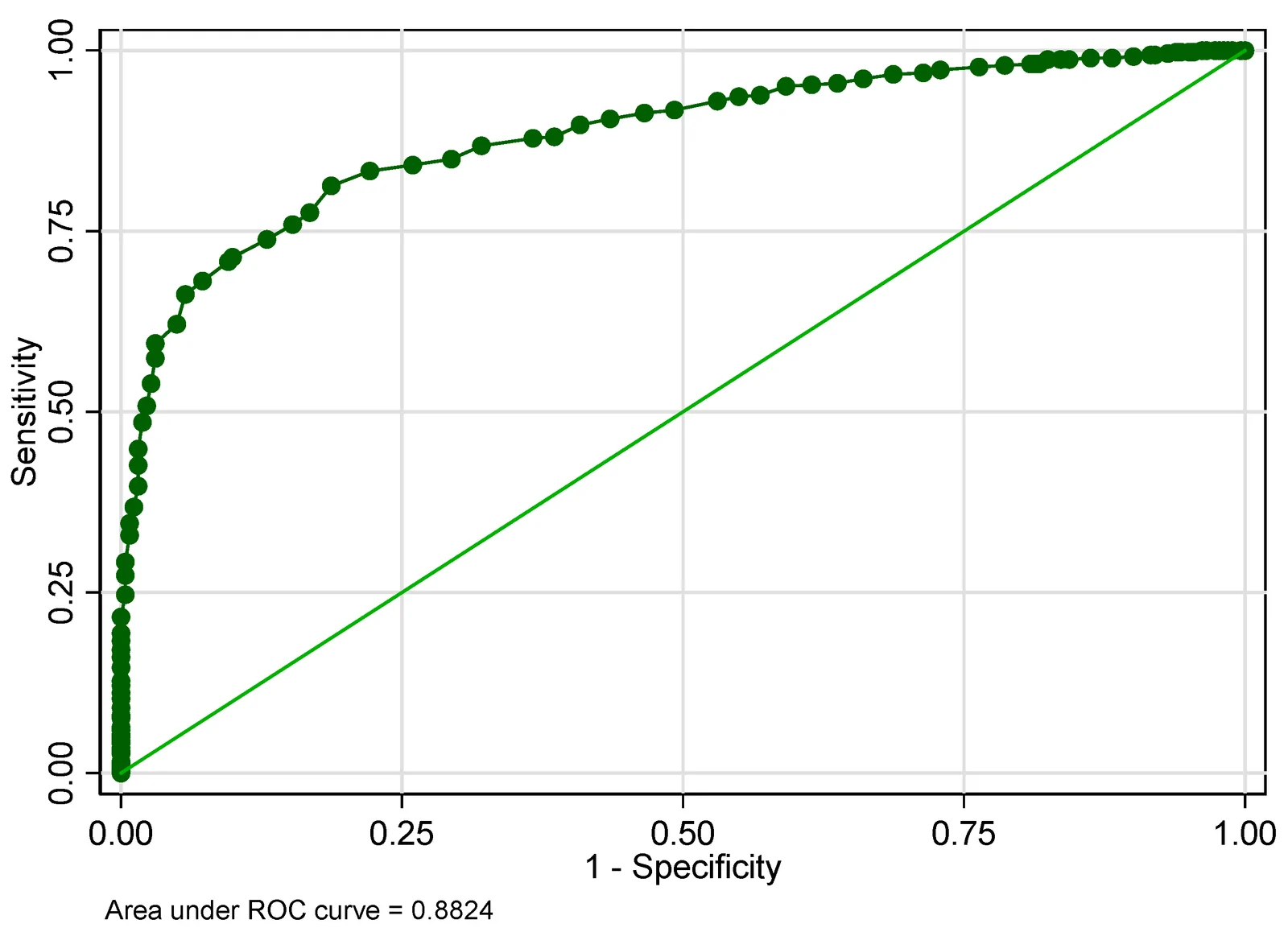
Receiver Operating Curve (ROC) comparing the capillary Hb measurements (HemoCue^®^ Hb 201^+^) to the venous Hb measurements (laboratory autoanalyzers).

**Table 1:** Sociodemographic characteristics of the study participants.

Characteristics	***n*** (%)
	*N* = 748
**Age**	
2–4 years	32 (4.28 )
5–11 Years	86 (11.50)
12–14 Years	43 (5.75)
≥15 years	587 (78.48)
**Gender**	
Male	323 (43.18)
Female	425 (56.82)
**Education level**	
Illiterate	31 (4.14)
Can read or sign	34(4.55)
Anganwadi/Preschool	41 (5.48)
School years 1 to 5	102 (13.64)
School years 6 to 10	253 (33.82)
Higher secondary schooling	130 (17.38)
Graduate and above	157 (20.99)
Occupation *	
Business/Trader/Shopkeeper/Self-employed	52 (6.95)
Government or Private job	108 (14.44)
Skilled Labour/menial workers	32 (4.28)
Homemakers/Pensioners/Senior citizens/Unemployed	280 (37.43)
Student	269 (35.96)
**History of chronic illnesses**	
No	647 (86.50)
Yes	101 (13.50)
**BMI (Kg/m^2^) (Mean ± SD)**	22.46 (5.59)

* Occupation not reported by 7 participants.

**Table 2:** Descriptive Statistics of venous and capillary haemoglobin measurements (*N* = 748).

Haemoglobin (g/dL)	Mean (SD) g/dL	Min–Max	Paired *t*-Test Mean Diff (SD)	*p* Value
HemoCue^®^ Hb 201^+^ Capillary Hb (On-site)	11.83 (1.95)	5.4–18	−0.73 (1.19)	<0.001
Haematology autoanalyzer Venous Hb	12.56 (1.89)	5.1–18.3		

**Table 3:** Measures of agreement between venous and capillary haemoglobin measurements (*N* = 748).

Bland–Altman Comparison			Concordance		
Mean Difference-Bias (95% CI)	Lower LoA (95% CI)	Upper LoA (95% CI)	Lin’s CCC (95% CI)	Standard Error	*p* Value
−0.73 (−0.82, −0.65)	−3.11 (−3.26, −2.97)	1.65 (1.51, 1.80)	0.75 (0.72,0.78)	0.015	<0.0001

Pearson Correlation Coefficient (precision) = 0.80; Bias Correction Factor (accuracy) = 0.93; Bradley–Blackwood F statistics = 142.04 (*p* ≤0.001); LoA: Limit of Agreement.

**Table 4:** Anaemia prevalence by venous and capillary samples.

Capillary Sample	Venous Sample			Test Performance Characteristics and Measures of Agreement
	Anaemia +	Anaemia −	Total	Sensitivity: 92.37% (95% CI: 90.46%, 94.27%) Specificity: 66.05% (95% CI: 62.66%, 69.44%) PPV: 59.46% (95% CI: 55.94%, 62.98%) NPV: 94.13% (95% CI: 92.45%, 95.82%) Kappa statistic: 0.52 (*p* value ≤ 0.001)
Anaemia +	242	165	407 (54.41%)	
Anaemia −	20	321	341 (45.59%)	
Total	262 (35.03%)	486 (64.97%)	748 (100%)	

PPV: Positive Predictive Value; NPV: Negative Predictive Value.

**Table 5:** Factors influencing the differences in haemoglobin measurements between venous and capillary blood samples.

Characteristics	Beta Coefficient 95% CI	*p*-Value	Adjusted Beta Coefficient 95% CI	*p*-Value
Age	0.014 (0.01, 0.02)	<0.001	0.013 (0.007, 0.02)	<0.001
Gender				
Female	−0.19 (−0.36, −0.02)	0.028	−0.29 (−0.47, −0.12)	0.001
Male	ref		ref	
BMI	0.04 (0.02, 0.05)	<0.001	0.02 (−0.002, 0.04)	0.074
History of chronic illnesses				
No	ref			
Yes	0.18 (−0.07, 0.42)	0.164		

## Data Availability

The data that support the findings of this study will be made available from the corresponding author upon request after March 2026 due to ongoing research activities related to the dataset.

## Supplementary Materials

The following supporting information can be downloaded at: https://www.mdpi.com/article/10.3390/diagnostics16203419/s1, Table S1: Measures of agreement between venous and capillary haemoglobin measurements in children up to 14 years of age; Table S2: Anaemia prevalence by venous and capillary samples in children up to 14 years of age; Table S3: Anaemia prevalence by venous and capillary samples in males 15 and above with venous haemoglobin between 8 and 13 g/dL; Table S4: Anaemia prevalence by venous and capillary samples in non-pregnant women 15 and above with venous haemoglobin between 8 and 13 g/dL.
