# Supplementary material for: Agreement Between Venous and Capillary Haemoglobin Concentrations in a High-Burden Urban Indian Setting: A Cross-Sectional Analysis
Source: Diagnostics (Basel). 2026 Oct 21;16(20):3419. doi: 10.3390/diagnostics16203419 (PMC13703079; doi:10.3390/diagnostics16203419)
Supplement: Supplementary file 1 [file diagnostics-16-03419-s001.zip › diagnostics-4041293-supplementary.pdf]

Supplementary Table S1: Measures of agreement between venous and capillary haemoglobin measurements in children up to 14 years of age

Mean (SD) of venous Hb samples	12.38 (1.19)
Mean (SD) of capillary Hb samples	12.15 (1.35)
Mean Difference- Bias (SD)	-0.22 (1.05)
95% Limit of Agreement (LoA)	-2.32, 1.87
95% CI of lower LoA	-2.60, -2.04
95% CI of upper LoA	1.59, 2.15
Lin's CCC, (95% CI)	0.65 (0.56,0.74)
Kappa coefficient (p value)	0.38 (<0.001)

Supplementary Table S2: Anaemia prevalence by venous and capillary samples in children up to 14 years of age)

Capillary sample	Venous sample			Test performance characteristics and measures of agreement
	Anaemia +	Anaemia –	Total	Sensitivity: 70.83% (95% CI: 63.81%, 77.85%)
Anaemia +	17	27	44 (27.33)	Specificity: 80.29% (95% CI: 74.15%, 86.44%)
Anaemia –	7	110	117 (72.67)	PPV: 38.64% (95% CI: 31.12%, 46.16%)
Total	24 (14.91%)	137 (85.09)	161 (100%)	NPV: 94.02% (95% CI: 90.35%, 97.68%)<br>Kappa statistic: 0.38 (P value = <0.001)

Supplementary Table S3: Anaemia prevalence by venous and capillary samples in males 15 and above with venous haemoglobin between 8 and 13 g/dL

Capillary sample	Venous sample			Test performance characteristics and measures of agreement
	Anaemia +	Anaemia –	Total	Sensitivity: 93.75% (95% CI: 86.05%, 101.45%)<br>Specificity: 33.33% (95% CI: 18.35%, 48.32%)<br>PPV: 88.24% (95% CI: 77.99%, 98.48%)<br>NPV: 50.00% (95% CI: 34.10%, 65.90%)<br>Kappa statistic: 0.31 (P value = 0.02)<br>Agreement= 84.21%
Anaemia +	30 (93.75)	4 (66.67)	34 (89.47)	
Anaemia –	2 (6.25)	2 (33.33)	4 (10.53)	
Total	32 (84.2)	6 (15.8)	38 (100%)	

Mean Difference- Bias = -0.87 (Limits of agreement: -3.43, 1.69)

Supplementary Table S4: Anaemia prevalence by venous and capillary samples in non-pregnant women 15 and above with venous haemoglobin between 8 and 13 g/dL

Capillary sample	Venous sample			Test performance characteristics and measures of agreement
	Anaemia +	Anaemia –	Total	Sensitivity: 94.27% (95% CI: 91.65%, 96.90%)<br>Specificity: 42.20% (95% CI: 36.62%, 47.78%)<br>PPV: 74.18% (95% CI: 69.24%, 79.12%)<br>NPV: 80.70% (95% CI: 76.24%, 85.16%)<br>Kappa statistic: 0.40 (P value = <0.001)<br>Agreement= 75.42%
Anaemia +	181 (94.27%)	63 (57.80%)	244 (81.06%)	
Anaemia –	11 (5.73%)	46 (42.20%)	57 (18.94%)	
Total	192 (63.79%)	109 (36.21%)	301 (100%)	

Mean Difference-Bias = -0.65 (Limits of agreement: -2.68, 1.38)
